# Effect of Media Use on HIV-Related Stigma in Sub-Saharan Africa: A Cross-Sectional Study

**DOI:** 10.1371/journal.pone.0100467

**Published:** 2014-06-19

**Authors:** Mesfin Awoke Bekalu, Steven Eggermont, Shoba Ramanadhan, Kasisomayajula Viswanath

**Affiliations:** 1 Center for Community-Based Research, Dana-Farber Cancer Institute, Boston, Massachusetts, United States of America; 2 Department of Social and Behavioral Sciences, Harvard School of Public Health, Boston, Massachusetts, United States of America; 3 School for Mass Communication Research, University of Leuven, Leuven, Belgium; 4 Department of Journalism and Communication, Bahir Dar University, Bahir Dar, Ethiopia; University of Toronto, Canada

## Abstract

It is known that HIV-related stigma hinders prevention efforts. Previous studies have documented that HIV-related stigma may be associated with socioeconomic and socioecological factors. Mass media use may moderate this association, but there is limited research addressing that possibility. In this study, based on cross-sectional data pooled from the 2006–2011 Demographic and Health Surveys of 11 sub-Saharan African countries (N = 204,343), we investigated the moderating effects of exposure to mass media on HIV-related stigma. Hierarchical regression analysis indicated that HIV-related stigma tends to be higher among rural residents and individuals with low levels of education and HIV knowledge, as well as those who do not know people living with HIV. Media use was generally associated with low levels of HIV-related stigma, and attenuated the gap between individuals with high and low educational levels. However, the effect of mass media was found to be stronger among urbanites rather than among rural residents, which could lead to a widening gap between the two groups in endorsement of HIV-related stigma. The implication of this study regarding the effect of media use on HIV-related stigma in sub-Saharan Africa is twofold: 1) mass media may have the potential to minimize the gap in HIV-related stigma between individuals with high and low educational levels, and hence future efforts of reducing HIV-related stigma in the region may benefit from utilizing media; 2) due perhaps to low media penetration to rural sub-Saharan Africa, mass media could have the unintended effect of widening the urban-rural gap further unless other more customized and rural-focused communication interventions are put in place.

## Introduction

HIV-related stigma is one of the principal factors that undermine public health efforts to combat the epidemic [1]. It is widely acknowledged that HIV-related stigma poses a significant threat to the effectiveness of prevention efforts targeted at both the general public and people living with HIV (PLH) [2]–[4]. It is significantly associated with depressive symptoms, greater HIV-related symptoms and poorer adherence to medication among PLH [5]–[7], and lack of utilization of HIV testing and maternity services in the general population [8], [9]. A systematic review assessing the role of HIV-related stigma in prevention efforts found that stigmatizing attitudes (held by the general public) and experiences of stigmatization (by PLH) are significantly associated with increased unsafe sexual practices, decreased utilization of biomedical-related prevention services (such as male circumcision, pre-exposure prophylaxis, microbicides and vaccines), use of prevention of mother-to-child transmission (PMTCT) practices, and utilization of HIV testing and antiretroviral treatment (ART) [10]. Among people who have never been tested for HIV, those with the tendency to endorse HIV-related stigma were more likely to engage in sexual risk practices and were less likely to get tested for HIV [2]. Specifically, in sub-Saharan Africa, the epicentre of the epidemic and the region that the present study addresses, several studies have shown that HIV-related stigma is associated with reduced levels of different HIV preventive practices, such as HIV testing [8], and ART and PMTCT utilization [3], [4] in both the general public and PLH.

Over the years, HIV-related stigma has drawn attention from numerous investigators in different prevention contexts [11]–[16]. Despite considerable efforts to reduce its prevalence and impact [17], stigma continues to be a major “road block” to HIV prevention efforts around the globe [18]–[20]. In sub-Saharan Africa, although studies suggest variability in the level of stigma by country [12], the problem remains a widespread concern across the region [20]. This prompts continued efforts to further understand the factors associated with HIV-related stigma and the threat it poses in different population sub-groups.

Consistent with research and theory in other health-related stigma, mainstream conceptualization of HIV-related stigma draws on Goffman’s seminal work and describes the phenomenon in terms of individuals who are seen as possessing ‘an attribute that is deeply discrediting’ [21], [22]. As such, Goffman’s ideas have mainly been used to study the psychological aspects of stigma, although his original formulation of stigma involved both psychological and social aspects of the stigmatizing process [23]. However, although this conceptualization has long dominated the field and unduly relegated the social and structural aspects of the problem [21], in recent years, a growing body of work has begun to suggest that HIV-related stigma is essentially a multi-level process that should be addressed at individual, familial and community levels [1], [24].

As a multi-level and multidimensional process, HIV-related stigma is associated with a range of factors. For instance, several studies have demonstrated associations between higher levels of economic development and lower levels of HIV-related stigma [25], [26],[27]. In a study of three African countries (Burkina Faso, Ghana and Zambia), Stephenson [25] observed that people living in communities with higher levels of male education or higher levels of male and female employment had significantly more supportive attitudes toward PLH. Other factors that are significantly associated with HIV-related stigma include HIV knowledge [14], [28]–[30], mass media exposure [31]–[33], socio-demographics such as age and gender [26], [29], [34], urban versus rural residence [29], [34], prior HIV testing experience [35] and knowing a PLH [29].

In sub-Saharan Africa, mass media have played a particularly significant role in alerting people about the infection, shaping societal norms and influencing behaviors associated with the transmission of the infection over the past several years [36]. However, although mass media are normally expected to inform or educate populations across the “masses”, there is mounting evidence of disparities in health communication among different social groups [37], [38]. Such disparities have been characterized as communication inequalities and their consequences have been elaborated in a model called the structural influence model (SIM) of health communication [39].

According to the structural influence model, communication inequality “may be defined as differences in the generation, manipulation, and distribution of information among social groups; and differences in (a) access and use, (b) attention, (c) retention, and (d) capacity to act on relevant information among individuals” ([39], p. 242). The model posits that the motivation for, access to, and use of health information and/or health-related media could at least partially explain the relationship between social determinants and health outcomes. Its premise is that “audiences attend and react to mediated content based on their structural location in the environment and the social roles they play at any given time” ([39], p. 244). The model contends that structural antecedents (e.g. SES and geography) determine both the information environment and the resources that are available for consumption and suggests that communication may have a role in linking social determinants with health outcomes including health cognitions, attitudes such as stigma and behaviors [39], [40]. Thus, in this study, we investigated whether exposure to mass media sources (radio, television and print) is associated with HIV-related stigma, and whether such exposure moderates the effects of education and urban versus rural residence on HIV-related stigma.

Drawing on the SIM, the present study attempted to examine whether differences in mass media exposure, as one form of communication inequality, moderates the associations between education and urban versus rural residence, on the one hand, and HIV-related stigma, on the other. Previous research in sub-Saharan Africa has shown that exposure to mass media sources is associated with higher socioeconomic status and urban residence, and such an exposure can both attenuate and widen the gap in HIV/AIDS-related outcomes such as HIV knowledge [37], [41] and condom use [41]. Despite some evidence of the overall association between mass media use and low levels of HIV-related stigma [31]–[33], little research has investigated whether differential mass media exposure moderates the relationships between HIV-related stigma and larger structural and socioecological factors such as education and urban versus rural residence. With this study, we seek to respond to this paucity of evidence and contribute to a growing body of evidence suggesting that communication inequality could be one of the factors that moderates the effects of background factors such as socioeconomic position and socioecological factors on health-related outcomes [37]–[41].

**Figure 1:**
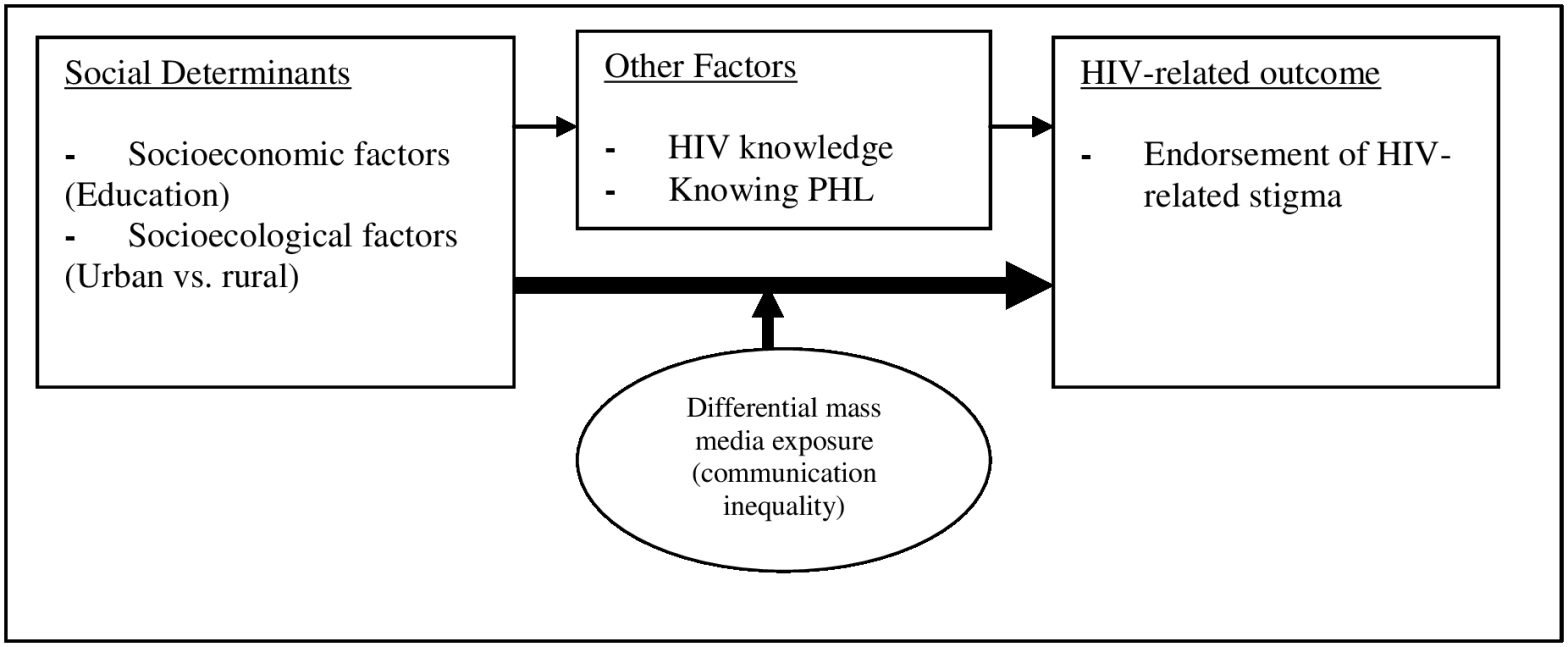
Conceptual framework of the study (adapted from the structural influence model, Viswanath et al., 2007).

The general heuristic framework of the study, adapted from the SIM (Figure 1), posits that HIV-related stigma may result from lack of awareness and/or relevant information about the pandemic (HIV knowledge) [42], overall educational attainment, and lack of real-life or vicarious experience with PLH. Knowing a PLH might be linked to the epidemiological context in which one lives; a person who lives in a high prevalence context may have a better chance of knowing a PLH than one who lives in a low prevalence context. In this regard, urban versus rural residence emerges as an important factor. In sub-Saharan Africa, HIV prevalence rates are generally higher in urban rather than in rural areas [43]. This prevalence imbalance could lead to urban-rural differences in direct experience with HIV/AIDS which in turn might be related to endorsement of HIV-related stigma. Additionally, drawing on the structural influence model of health communication [39], the model proposes that the effects of education and place (urbanity versus rurality) on HIV-related stigma, which may be partly mediated through HIV knowledge and knowing a PLH, could be moderated by exposure to mass media sources (see Figure 1).

## Methods

### Dataset

Data for this study come from the Demographic and Health Surveys (DHS) database. The DHS surveys are nationally representative, have relatively large sample sizes (usually between 5,000 and 30,000 households), and are typically conducted about every 5 years, to allow for comparisons over time [44]. DHS recruits respondents using a multi-stage sampling procedure that stratifies all the states of each country into urban and rural areas, with each state’s sample size determined based on the size of the urban and rural populations as well as each state’s gender ratio [41]. Every age-eligible woman (age 15–49) and man (age 15–64) in the selected households are interviewed using individual questionnaires. For this study, data have been drawn from the most recent surveys (2006–2011) of eleven countries representing the different (East, West, Central and South) regions of sub-Saharan Africa (N = 204,343). Table 1 summarizes the basic demographic characteristics of the respondents.

**Table 1:** Demographic characteristics of respondents in the eleven countries.

Demographics		Country										
		East		West					Central	South		
		Ethiopia (’11)	Uganda (’06)	Benin (’06)	Mali (’06)	Niger (’06)	Nigeria (’08)	Sierra Leone (’08)	DR Congo (’07)	Lesotho (’09)	Swaziland (’07)	Zambia (’07)
Gender	Male	46.10%	22.70%	23.00%	22.40%	27.80%	31.70%	30.80%	32.20%	30.30%	45.50%	47.60%
	Female	53.90%	77.30%	77.00%	77.60%	72.20%	68.30%	69.20%	67.80%	69.70%	54.50%	52.40%
Age	15–19	21.80%	22.90%	17.40%	21.20%	19.80%	18.70%	17.00%	20.40%	24.50%	27.60%	22.10%
	20–24	17.50%	18.70%	16.40%	17.40%	17.10%	17.40%	15.10%	20.90%	20.00%	20.80%	18.10%
	25–29	17.80%	16.00%	19.00%	16.20%	17.10%	17.90%	18.90%	15.90%	15.20%	15.00%	17.30%
	30–34	12.30%	14.40%	15.00%	13.00%	13.30%	13.50%	13.60%	13.50%	12.20%	11.80%	14.50%
	35–39	11.50%	11.60%	12.20%	11.80%	11.80%	11.60%	15.10%	10.40%	9.50%	9.90%	10.70%
	40–44	8.20%	8.60%	9.10%	9.50%	9.70%	9.10%	9.30%	8.90%	7.90%	7.90%	7.40%
	45–49	6.70%	6.90%	7.80%	8.20%	7.80%	8.40%	8.00%	7.20%	7.80%	7.00%	6.30%
	50–54	2.40%	NA	1.50%	1.60%	2.00%	1.90%	1.70%	1.70%	1.50%	NA	2.10%
	55–59	1.70%	NA	0.90%	1.10%	1.40%	1.40%	1.30%	1.20%	1.50%	NA	1.50%
	60–64	NA	NA	0.80%	NA	NA	NA	NA	NA	NA	NA	NA
	DK	NA	1.10%	NA	NA	NA	NA	NA	NA	NA	NA	NA
Education	No education	41.60%	17.40%	58.80%	73.80%	72.10%	34.60%	57.70%	16.40%	5.60%	8.10%	7.60%
	Incomplete primary	35.90%	48.50%	19.10%	11.00%	12.60%	6.20%	9.80%	28.40%	30.50%	23.60%	30.90%
	Complete primary	5.00%	10.70%	2.80%	2.20%	2.00%	13.90%	4.10%	6.90%	20.00%	9.90%	18.90%
	Incomplete secondary	7.80%	17.80%	16.60%	11.30%	11.00%	18.30%	20.90%	36.00%	32.00%	42.80%	28.50%
	Complete secondary	2.00%	1.10%	1.10%	0.70%	0.90%	17.30%	3.90%	8.00%	7.10%	7.00%	7.80%
	Higher	7.70%	4.60%	1.50%	1.10%	1.50%	9.70%	3.70%	4.40%	4.80%	8.50%	6.20%
Residence	Urban	31.20%	16.70%	42.00%	35.60%	37.20%	32.00%	42.80%	47.30%	24.80%	32.60%	44.00%
	Rural	68.80%	83.30%	58.00%	64.40%	62.80%	68.00%	57.20%	52.70%	75.20%	67.40%	56.00%
**Total N**		**30625**	**11034**	**23115**	**18790**	**12772**	**48871**	**10654**	**14752**	**10941**	**9143**	**13646**

### Measures

DHS utilizes standard measures across countries to allow for merging and/or comparison of data. The measures undergo psychometric testing and translation checks, and are widely accepted. For this paper, we included the measures for one dependent, five independent and two control variables.

#### Dependent variable

*HIV-related stigma*. Four binary measures were used to assess respondents’ attitudes towards people living with HIV. Respondents were asked: 1) if they would care for a relative who is sick of AIDS in their own households, 2) if they would want to keep a family member’s HIV positive status secret, 3) if they would be willing to buy fresh vegetables from a market vendor who is HIV positive, and 4) if they thought a female teacher who is HIV positive but not sick of AIDS should be allowed to continue teaching. Items 1, 3, and 4 were inversely coded so that agreement to one or more of these statements could show endorsement of HIV-related stigma.

#### Independent variables

*Educational attainment*: Respondents were asked: a) if they have ever attended school and b) what their highest level of schooling was, with six resulting categories: no education (0), incomplete primary (1), complete primary (2), incomplete secondary (3), complete secondary (4), and higher than secondary (5).

*Place of residence*: This variable represents urban versus rural residence; urbanity was coded 1, and rurality 2.

*Media use*: Respondents were asked the frequency of using print media, radio and television, with three response options: not at all (0), less than once a week (1), and at least once a week (2).

*Knowing PLH*: This variable was measured by asking respondents if they knew someone who is, or is thought to be, living with HIV.

*HIV Knowledge*: This measure assessed respondents’ transmission and prevention knowledge. Respondents were considered knowledgeable about HIV transmission if they indicated that: 1) mosquito bites and 2) sharing food with people with the virus could not spread the infection, and that 3) it was possible for a healthy-looking person to have HIV. Respondents were considered knowledgeable about HIV prevention if they responded affirmatively that one could reduce the risk of contracting the virus if: 1) they abstained from sexual intercourse, 2) had only one uninfected sexual partner, and 3) used condoms consistently.

#### Control variables

Respondents’ current age and gender have been included in the analysis as control variables.

#### Ethical Statement

DHS is an open data collection activity whose aims and procedures are clear to the governments of all participating countries. Its data collection instruments are, to large extent, standardized and are widely accepted. The broad goals of the exercise are explained to each respondent by fieldworkers during household visit [45]. As such, confidentiality is ensured and ethical issues are duly heeded in all DHS surveys. MEASURE DHS’ guideline also stipulates that “the DHS surveys are anonymous surveys which do not allow any potential identification of any single household or individual in the data file.” ([44], p. 3).

### Statistical Analysis

Using SPSS 20, a hierarchical regression analysis was performed to determine whether educational attainment, urban versus rural residence, HIV knowledge, knowing a PLH and media use are associated with HIV-related stigma. An analysis of interaction terms was performed to determine whether media use moderates the effects of educational attainment and place of residence on HIV-related stigma. Based on our study objectives, the variables were entered into the regression model in a series of steps. First, the age and gender of the participants were entered as a control block into Block 1, HIV knowledge and knowing PLH were entered into Block 2, and educational attainment and urban versus rural residence were entered into Block 3. Because we were interested in the individual main effect of media use, this variable was entered into Block 4; the two interaction terms (media use x education, media use x urban versus rural residence) were entered into Block 5. To reduce potential problems of multicollinearity, all of the independent variables were centered by subtracting the means from each score before forming the interaction terms.

## Results

**Figure 2:**
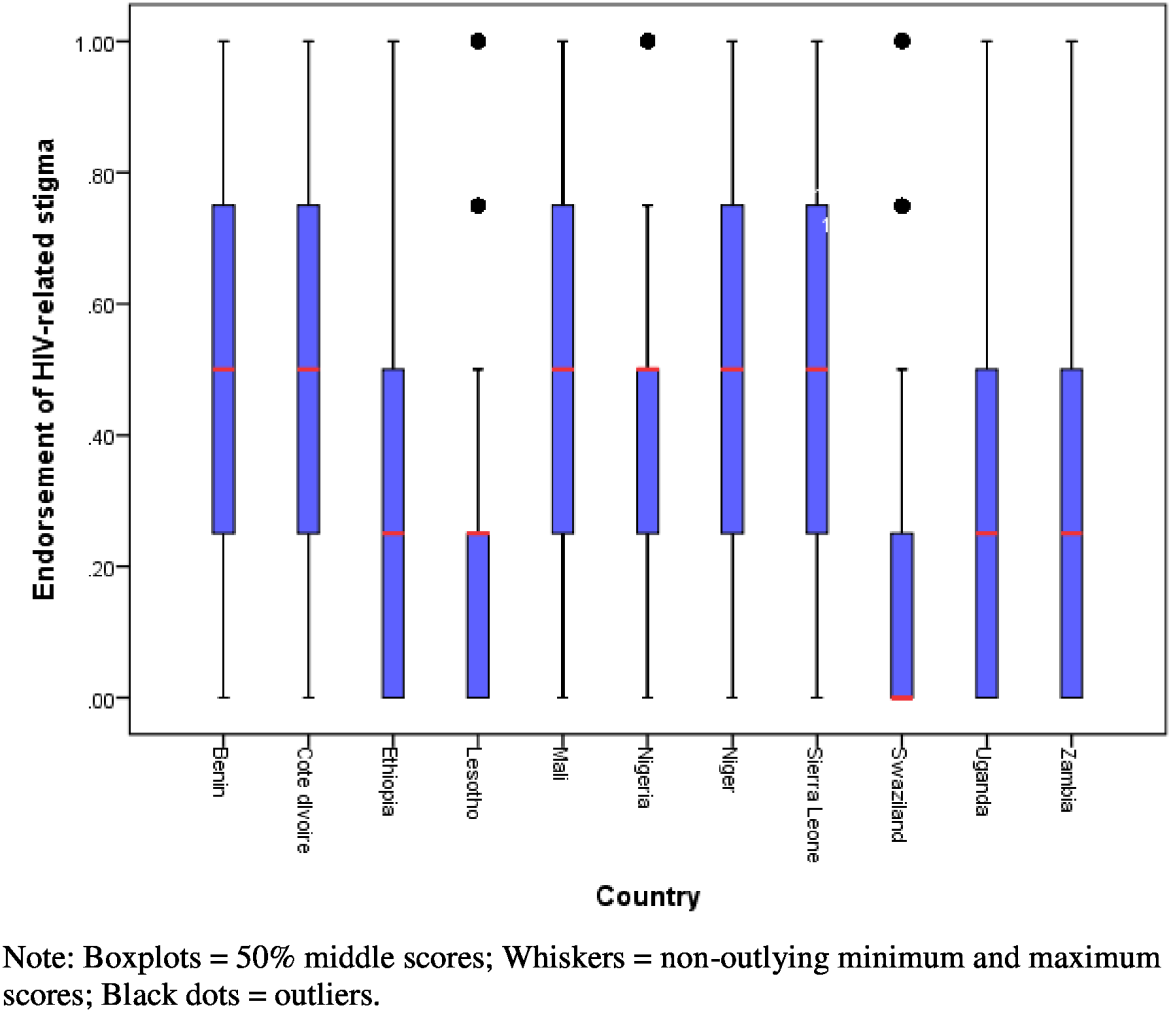
Level of HIV-related stigma endorsement in 11 sub-Saharan African countries.

Preliminary analysis indicated that there is variability in endorsement of HIV-related stigma within and between countries. As visualized in Figure 2, in 8 of the 11 countries, endorsement of stigma ranges between 0 (no stigma) and 1 (endorsement of all the four stigma items). Stigma appears to be of moderate magnitude in western and central African countries (Benin, DR Congo, Mali, Niger, Nigeria and Sierra Leone), whereas it is of low magnitude in eastern and southern African countries (Ethiopia, Lesotho, Swaziland, Uganda and Zambia).

Zero-order bivariate correlations showed that the independent variables are significantly associated with one another and the outcome variable (Table 2). All the tested variables and the two interaction terms emerged as significant predictors (Table 3). Block 1– the control block – accounted for 1.8% of the total variance of the outcome variable (*p*<0.0001), with gender being a significant predictor (β = .13, *p*<0.0001). Controlling for age and gender, HIV knowledge (β = −0.28, *p*<0.0001) and knowledge of a PLH (β = −0.15, *p*<0.0001) explained an additional 11.1% of the variance (*p*<0.0001), with HIV knowledge making the highest contribution. Moreover, educational attainment (β = −0.21, *p*<0.0001) and urban versus rural residence (β = 0.02, *p*<0.0001) were significantly associated with HIV-related stigma, explaining additional 4% of the variance. Media use also emerged as a significantly associated factor, explaining an additional 0.3% of the variance (β = −0.07, *p*<0.0001) after controlling for demographics (age and gender), HIV knowledge, knowledge of a PLH, educational attainment and place of residence (see Table 3).

**Table 2:** Zero-order bivariate correlations between the independent and dependent variables.

	1	2	3	4	5	6
1. Urban/rural	1					
2. Education	−.342**	1				
3. HIV knowledge	−.188**	.363**	1			
4. Media Use	−.407**	.569**	.334**	1		
5. Knowing PLH	−.025**	.147**	.172**	.119**	1	
6. HIV-related stigma	.160**	−.316**	−.313**	−.257**	−.198**	1

**Correlation is significant at the 0.01 level (2-tailed).

**Table 3:** Summary of results from the hierarchical regression analysis.

Variable	β	*t* Value	ΔR^2^ (%)	*F* Change
Block 1				
Age	−.005	−1.76		
Gender (female: high)	.13*	48.69	1.8*	1213.08
Block 2				
HIV Knowledge	−.28*	−107.38		
Knowing PLH	−.15*	−55.86	11.1*	8558.22
Block 3				
Education	−.21*	−75.97		
Urbanity vs. rurality (rural: high)	.02*	5.86	4.0*	3244.96
Block 4				
Media use	−.07*	−20.72	.3*	429.21
Block 5				
Media use × education	.01*	4.34		
Media use × urbanity vs. rurality	.02*	7.28	0	29.40

*p<0.0001.

**Figure 3:**
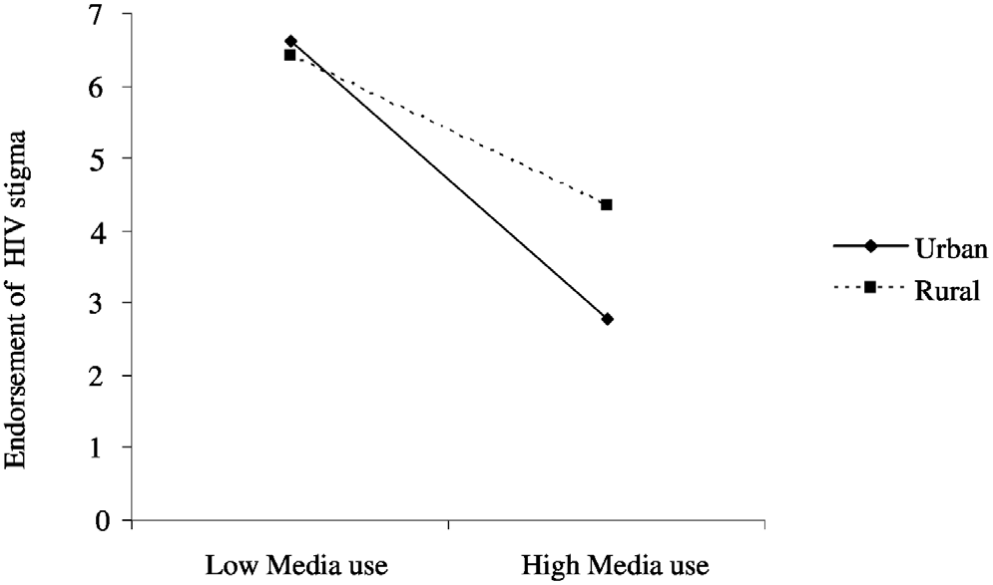
Regression plot for the interaction between media use and urbanity vs. rurality.

Moreover, the interaction terms showed that there are significant interactions between media use and two of the three predictors. Media use significantly moderated the effects of urban versus rural residence on HIV-related stigma (β = 0.02, *p*<0.001). As Figure 3 shows, endorsement of HIV-related stigma was generally negatively associated with media use in both urban and rural groups. However, the gap between the two population sub-groups is likely to increase as media use increases (see Figure 3).

**Figure 4:**
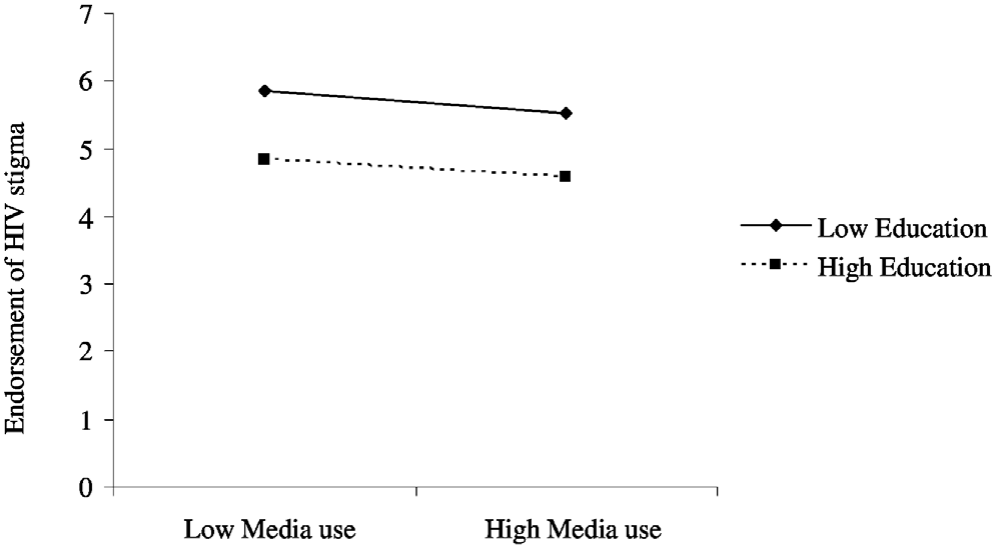
Regression plot for the interaction between media use and education.

The interaction of media use with educational attainment was also significant (β = 0.01, *p*<0.001). Plotting the regression coefficients yielded an ordinal interaction plot visualized in Figure 4. As media use increases, endorsement of HIV-related stigma shows the tendency of decreasing in both individuals with high and low educational attainment. Moreover, media use attenuates the gap in endorsing HIV-related stigma between the two social groups (see Figure 4).

## Discussion

This study identified some of the factors that are associated with HIV-related stigma and examined the role of media use in moderating the effects of education and place of residence on HIV-related stigma in 11 sub-Saharan African countries. The data suggest that although HIV-related stigma tends to be of low to moderate level in the sample countries, there is variability within and between countries. The within-country variability highlights differing levels of HIV-related stigma among population sub-groups. We also found variations between western and central African countries (moderate level of endorsement of stigma) and eastern and southern African countries (low levels of endorsement of stigma). HIV-related stigma tends to be more pervasive in countries with low rates of HIV prevalence than in countries with high prevalence. Recent epidemic updates from UNAIDS [46] indicate that although sub-Saharan Africa continues to be the epicentre of the pandemic, much smaller proportions of the population are living with HIV in western and central African countries than eastern and southern African countries.

Despite its variability, HIV-related stigma was significantly associated with a few key factors. Consistent with previous studies that have demonstrated that HIV/AIDS-related outcomes such as HIV knowledge and condom use are positively associated with people’s socioeconomic status in sub-Saharan Africa [41], the present study has found that HIV-related stigma tends to be higher among people with low educational attainment than among those with high educational attainment. Similarly, stigma was found to be higher among rural residents, people who have low levels of HIV knowledge, and people who do not know a PLH than among urban residents, people with high levels of HIV knowledge, and those who know a PLH. Moreover, we found that stigma tends to be higher among individuals with low media exposure than among those with high media exposure.

The relative importance of the associations between each of these factors and HIV-related stigma can be gathered from the amount of variance explained in each block of the regression model. As indicated in Table 3, HIV knowledge and knowing a PLH followed by education and urban versus rural residence substantially explained variability in HIV-related stigma. It should also be noted that although these factors have emerged as significant predictors of stigma across the eleven sub-Saharan African countries, the vast majority of variance in HIV-related stigma remained unaccounted for. This could be due to several reasons. First, the number of factors we included in our model was relatively limited. Although the SIM, the theoretical model based on which we developed our conceptual framework, contains a number of socio-structural, demographic and communication variables, we only dealt with a few key variables which were available in the DHS datasets. Second, although DHS provides a rich set of data on multiple countries, some of its measures are quite limited. Specifically, we would like to note that the measure of stigma is limited vis-à-vis the multi-level and multidimensional nature of the phenomenon and this is likely to have limited the possibility of capturing robust relationships. Third, the findings could also suggest the presence of other importantly associated factors that might be of local or regional nature in the sampled countries. Clearly, further studies utilizing a more qualitative approach are warranted in the different countries to explicate micro, meso and macro level factors associated with HIV-related stigma.

The findings of this study provide further evidence to the conclusions of previous studies that have reported disparities in different HIV/AIDS-related outcomes, such as HIV knowledge, condom use and the utilization of HIV testing services between population sub-groups [37], [41]. The concept of *intersectionality* [47] highlights additional potential implications of these findings. The intersectional approach posits that health disparities cannot be attributed to one or more of the social determinants of health but instead lie on the intersection of multiple factors that are interwoven in a complex and reactive manner [47]. According to this approach, research and interventions on health disparities need to focus on the intersection where multiple identities of social groups converge [48]. The present study has found that HIV-related stigma, which has long been considered a hurdle for prevention practices, is associated with illiteracy, rurality, lack of relevant knowledge about the epidemic, and lack of exposure to mass media sources. As such, the social location in which this attitude prevails might well be visualized at the intersection of these multiple identities. Likewise, the fact that stigma is high in these communities implies that PLH living in such communities could experience high levels of stigma. Thus, the intersectional conceptualization of HIV-related stigma can also represent the situation of those stigmatized. Previous research in other contexts has indeed found that the situation of HIV-positive women with multiple identities of marginalized social status, racism and sexism can well be described by an intersectional model of stigma and discrimination [1]. This implies that interventions targeted at reducing HIV-related stigma in sub-Saharan Africa may only be successful to the extent that they focus on the intersection of these multiple factors rather than any one of the individual factors.

The study has also found that higher exposure to mass media sources is associated with lower level of HIV-related stigma in both high and low education sub-groups. Indeed, the gap between the two groups has tended to attenuate as exposure to mass media sources increases, suggesting the mainstreaming role of mass media [49]. In other words, segments of the population with high levels of exposure to mass media sources are more likely to have similar levels of HIV-related stigma, regardless of differences in educational background. On the contrary, although HIV-related stigma has generally tended to decrease as exposure to mass media sources increased in both urban and rural groups, urbanites benefited more from mass media than did their rural counterparts, leading to a widening gap between the two population sub-groups. Overall, the findings of the present study suggest that despite their capacity to generally reduce HIV-related stigma in the total population, mass media can both narrow and widen gaps in stigma between population sub-groups. While the gap between individuals with high and low education was more likely to be narrow, the gap between urban and rural residents was more likely to be widened.

The findings may have several implications for HIV-related stigma reduction campaigns in sub-Saharan Africa. First, the fact that HIV knowledge is substantially associated with HIV-related stigma clearly speaks to the need to leverage ongoing HIV/AIDS awareness raising and education programs in the region to further counteract stigmatic attitudes at individual and societal levels. Second, because people who know a PLH are more likely to have reduced levels of HIV-related stigma, it could be argued that ongoing and future stigma reduction interventions in the region might well benefit from actively involving HIV positive individuals in their prevention efforts. This strategy, known as prevention-with-positives (PwP), has indeed been acknowledged in other prevention contexts [50]. By actively involving PLHs in mass media HIV/AIDS programs and/or giving them access to mainstream media, a form of vicarious experience with PLHs could also be provided to the general public. Nevertheless, the long term impacts of such strategies will largely depend on the extent to which the field of HIV-related stigma reduction becomes broader and targets multiple stigma domains at multiple levels [51].

Moreover, in areas where mass media are available and/or accessible, the findings suggest that mass media information campaigns could be harnessed to address possible communication inequalities between certain segments of the population and thereby reduce the gap in endorsing HIV-related stigma. Specifically, stigma among individuals with low levels of education can possibly be addressed using mass media. The fact that there is an urban-rural gap in HIV-related stigma across sub-Saharan Africa suggests that the rural population, which accounts for over 60% of the region’s total population, will be increasingly at risk if they continue to stigmatize PLHs and fail to engage in open talks about the dangers of the pandemic. Although mass media would normally be expected to narrow the urban-rural gap, the reality on the ground appears to be the opposite. Efforts should therefore be made to compensate for the relative lack of exposure to mass media among rural residents. For instance, community-based participatory programs utilizing existing social, cultural and religious networks might be useful [52]. Additionally, although the urban-rural gap which is bound to widen as media use increases might more generally be explained in terms of low mass media penetration to the rural areas of sub-Saharan Africa, factors related to rural people’s information processing capacity and mass media messages’ clarity and relevance to the rural audiences should also be heeded. The broader literature on health communication in sub-Saharan Africa suggests that mainstream health information systems in most countries in the region leave many of the health information needs of rural people largely unmet [53]. Previous studies have also shown that the rural people tend to have problems receiving and understanding HIV/AIDS messages, a significant dimension of communication inequality, and thus, researchers and interventionists must acknowledge socio-cultural factors in planning health information interventions targeting rural areas [53], [54]. Accordingly, interventions that are more sensitive to community-level factors might be useful for addressing the observed urban-rural disparities.

Any generalization and/or application of findings presented here must take into account the limitations of the study. First, although the study pooled nationally representative data from multiple sub-Saharan African countries, the cross-sectional nature of the data needs to be taken into consideration. Second, the fact that the data have been gathered at different time-points (2006–2011) may need to be taken into account, although we have used the most recent datasets of each country in which our variables of interest were found. Lastly, although the study represents a novel application of communication inequality to the realm of HIV prevention in sub-Saharan Africa, the fact that the DHS media use measures are basic communication measures that do not specifically capture levels of exposure to HIV/AIDS-related media content stands out the main limitation of the present study. Whereas further studies utilizing experimental designs are necessary to validate the conclusions drawn from this cross-sectional study, we believe that the findings provide important insights regarding the role exposure to mass media sources plays in the arena of HIV prevention in sub-Saharan Africa in general and may also be useful to aid ongoing and future HIV-related stigma reduction communication campaigns in the region.
